# Childhood Use of Coin Pusher and Crane Grab Machines, and Adult Gambling: Robustness to Subjective Confidence in a Young Adult USA Sample

**DOI:** 10.1007/s10899-023-10261-8

**Published:** 2023-10-25

**Authors:** Oliver Bastiani, Alex M. T. Russell, Philip Newall

**Affiliations:** 1 School of Psychological Science, University of Bristol, 12a Priory Road, Bristol, BS8 1TU UK; 2 Experimental Gambling Research Laboratory, School of Health, Medical and Applied Sciences, CQUniversity, 400 Kent St, Sydney, NSW 2000 Australia

**Keywords:** Penny Falls, Penny Pushers, claw Cranes, Youth Gambling, Child Gambling

## Abstract

Gambling as a youth is a risk factor for experiencing gambling-related harm as an adult. Most youth gambling research focuses on illegal engagement with age-restricted products, but youth can also gamble legally, by for example betting with friends, or via coin pusher and crane grab machines. Research has associated recollected rates of usage of these machines as a child with adult gambling participation and problems, but only in the UK and Australia, and has not tested for robustness to subjective confidence. The present study conceptually replicated these prior studies by investigating the association between recollected childhood use of coin push and crane grab machines, and adult gambling behavior, in a young adult USA sample. Participants rated their subjective confidence to test if individual differences in recollection biases provided a better account for any observed associations. Results found high recollected engagement rates for both coin pusher (87.2%) and crane grab machines (97.0%), and 5 of the 6 tested associations between youth machine usage and adult gambling engagement and problems were significant and in the hypothesized direction. Rates of subjective confidence were on average high (83.3 and 89.2 on a 0 to 100 scale), and generally did not interact with participants’ recollected rates of machine use. These findings extend prior research on potential public health concerns around children’s legal engagement with coin pusher and crane grab machines to a new country, the USA.

Most youth gambling research focuses on the illegal use of age-restricted gambling machines and services (Calado et al., 2017; Derevensky & Gilbeau, 2015; Frank, 1990; Ho, 2017; Olason et al., 2011). But youth can also gamble legally, such as by betting privately with friends (Hing et al., 2021), or by buying “gambling-like” video game loot boxes (Xiao et al., 2022). Coin pusher machines (*aka* “penny falls”, “penny pushers”) and crane grab machines (*aka* “claw cranes”) are two commercial products that also allow youth to gamble legally, and which have been relatively under-researched internationally. Coin pusher machines involve inserting coins which fall onto a mechanised platform which pushes coins towards a drop, with the objective of winning more coins than inserted (or small toys which rest on the coins) in the machine. Crane grab machines involve monetary payments which facilitate the chance to grab a prize of greater value by using an overhead claw crane. While the cost of playing these machines and the value of their winnings are relatively small, they both meet formal definitions of gambling as they involve the voluntary risking of money in pursuit of the hope of winning something of greater value (Neal et al., 2005). A recent study of UK children aged 11–16 found that 22% had spent their own money on at least one of these machines within the last 12 months, compared to 1% who had gambled online (Gambling Commission, 2022). These figures demonstrate that a public health approach toward youth gambling harm should consider the potential risks of coin pusher and crane grab machines.

Researchers have highlighted these two machines for some years (Carran & Griffiths, 2015; Holmes, 1988; Miers, 2013; Wardle, 2021), but quantitative empirical research has only begun to emerge recently (Newall et al., 2020a, b, 2021). For example, one recent study on 2,000 UK young adults aged 19–24 years old showed that 94.9% recollected ever using coin pusher, and 93.0% recollected ever using crane grab machines as a child (Parrado-González & Newall, 2023). This study added to potential concerns around the high current rates of childhood usage of these machines (Gambling Commission, 2022), as five out of seven tested associations suggested that childhood usage of these machines was generally predictive of adult gambling participation and problems (Parrado-González & Newall, 2022). However, to date only one study has been done outside the UK, which showed that 55.9% and 89.2% of an Australian adult sample recollected using coin pusher and crane grab machines, respectively (Newall et al., 2021). This Australian study used an older (*M* = 30.9) and smaller (*N* = 640) sample than the UK-based research, suggesting that more international research is still needed. Such research is important, as illegal gambling as a youth is a risk factor for experiencing gambling-related harm as an adult (Jiménez-Murcia et al., 2010), and youth problem gambling prevalence rates often exceed rates seen in adults (Calado et al., 2017; Derevensky & Gupta, 2000). Furthermore, the associations with adult gambling problems observed in legal childhood gambling may indicate that legal gambling as a youth is also a risk factor for adult gambling-related harms. Problem gambling is an issue as it can cause harm for gamblers themselves (Chowdhury et al., 2017; Langham et al., 2015; Wardle et al., 2019), as well as those around them (Chowdhury et al., 2017; Kourgiantakis et al., 2013), and disproportionately affects marginalised and disadvantaged groups (Abbott, 2020; Rintoul et al., 2013). These harms are echoed in youth problem gambling, which is associated with depression and suicidal behaviour (Derevensky & Gupta, 2004). If the onset of problem gambling behaviours can be observed or predicted during youth, such research could inform interventions which may target and prevent gambling-related harms in the future.

This evidence linking recollected childhood machine usage and adult gambling is limited by the retrospective nature of childhood machine usage (Newall et al., 2020a, b, 2021; Parrado-González & Newall, 2022). Firstly, participants may be experiencing general memory biases which may distort the accuracy of their recollection (Lacy & Stark, 2013). However, even the collection of young adult samples cannot account for plausible individual differences in memory recall across the sample. Problem gambling involves cognitive distortions affecting memory, such as hindsight bias and selective memory (Russell et al., 2019; Toneatto, 1999). It is plausible that problem gamblers may better recollect their usage of coin pusher and crane grab machines as children, perhaps because these early experiences are more consistent with their current identity as gamblers. This account could plausibly explain the observed higher rates of childhood machine usage among adult problem gamblers. Getting participants to answer a measure of subjective confidence in their recollected level of childhood machine usage is one way to gauge whether individual differences in (conscious) recollection biases are driving these results.

To the best of our knowledge, there is no prior evidence on the use of these machines and links with adult gambling engagement and problems in the USA. However, the USA offers an interesting comparison to countries previously studied, due to potentially different attitudes to gambling. For example, in the UK and Australia, a product is considered to be gambling if there is any element of chance in determining the outcome, no matter how much skill may also be involved. In many states in the USA, if skill is more important than chance in determining the outcome (the Dominant Factor Test), the product is not considered gambling (Schiavone, 2010; Tselnik, 2006). Until 2018, sports betting was not allowed in most parts of the USA (“Murphy v. National Collegiate Athletic,“ 2018), while it is commonplace in the UK and Australia, and has been legal in Australia since the 1980s (Australian Institute for Gambling Research, 1999). These examples highlight some of the differences between the USA and the other countries where this research has taken place, and why the USA is of interest for further research.

Therefore, the present study is a conceptual replication of Parrado-González and Newall (2022). The present study recruited participants who were born in and resident in the USA, in order to uniquely assess rates of recollected machines usage (in a non-representative sample) and associations with adult gambling in a country that is culturally-similar, but that also has some distinct gambling contexts. Confidence judgements were also included to measure participants’ confidence in their childhood recollections, and to test for potential interaction effects that could indicate whether any observed main effects could be explained by individual differences in recollection biases.

The following hypotheses were tested:

H1. That any level of recollected engagement with a given machine, versus not recollecting using a machine, is associated with being an adult gambler.

H2. That for adults recollecting a given machine, higher frequencies of machine use recalled are associated with being an adult gambler.

H3. That any level of recollected engagement with a given machine, versus not recollecting using a machine, is associated with higher PGSI scores among adult gamblers.

H4. For adult gamblers who recollect using a given machine, that higher frequencies will be associated with higher PGSI scores.

H5. That in models similar to those in H1-H4, but adding a main effect and an interaction for subjective confidence, that the resulting interaction effects will be nonsignificant. Negative interactions, whereby there are stronger associations for people who are less confident, would be the most likely to indicate that the present results are not robust to memory biases across the sample.

## Methods

Ethical approval was obtained for this study from the University of Brisol School of Psychological Science Research Ethics Committee (#13,776). Data, materials, and the preregistration document are available from https://osf.io/kmy92.

### Participants

An even split between female and male participants who were US residents and who were also born in the USA were recruited for this study. Participants were aged between 19 and 24 years. Recruitment took place via the online recruitment platform Prolific, where participants were paid $0.50 (mean duration = 1.6 min; $18.75 per-hour pro-rata). Samples recruited via Prolific are more diverse than and arguably provide better data quality than undergraduate samples (Douglas et al., 2023).

Our plan was to obtain usable responses from 2,000 participants, as this was the sample size that had been used in two previous studies on the topic (Newall et al., 2021; Parrado-González & Newall, 2022). Given that the below-described seriousness check which was expected to lead to the loss of a small proportion of collected responses, we collected data from 2,063 participants initially. Overall, 47 participants were excluded due to failure of the seriousness check, and a further 11 were excluded due to missing age data, leaving a sample of 2,005 participants for analysis. This sample consisted of 48.6% male and 51.4% female participants, ranging from 19 to 24 years (*M* = 21.9, SD = 1.6). Of these participants, 423 were between the ages of 19 and 20, and 1,582 were aged 21 or older.

### Materials

*Childhood machine use*: Participants were presented with an image and short description of a given machine (either a coin pusher or crane grab machine, on separate pages). Underneath this was the question “How often do you remember using (either coin pusher or crane grab) machines **while being under the age of 18?**“, with a 5-point scale to select an answer from: Never, Seldom, Occasionally, Frequently or Very Frequently (Parrado-González & Newall, 2022). Participants were then asked the question: “How confident are you in your recollection of this?”. A slider from 0 to 100 was used to answer this question, with 0 marked as “not at all confident” and 100 marked as “very confident”.

#### Adult Gambling

Participants were asked if they had engaged in any monetary gambling in the last 12 months. Consistent with the methodology used in gambling prevalence surveys (Sturgis & Kuha, 2022), only participants who responded “yes” were then asked to complete the Problem Gambling Severity Index (PGSI), the most commonly used scale for problem gambling (Ferris & Wynne, 2001).

### Procedure

Participants completed the adult gambling and childhood machine usage blocks in random order. As a data quality check, participants then completed a self-reported carelessness check: “In your honest opinion, should we use your data in our analyses in this study? (Do not worry, this will not affect your payment, you will receive the payment code either way.)” (Brühlmann et al., 2020). All participants that responded “no, please do not use my data” were excluded from analysis (*n* = 47). This was the only data quality check performed on the data. Demographic data were automatically collected by Prolific.

### Statistical Analysis

For hypotheses 1 and 2, the dependent variable for each product was whether the participant had gambled in the past 12 months. Therefore, analysis (1) and (2) used logistic regression, with the participant’s adult gambler status as the dependent variable. For hypotheses 3 and 4, the dependent variable for each product was the Problem Gambling Severity Index (PGSI), which tends to be heavily positively skewed. Analysis (3) and (4) were therefore planned using negative binomial models, which can account for this skewness (Welte et al., 2004). (PGSI scores exhibited a large number of zeroes, and so zero-inflated negative binomial models were also tested as an exploratory analysis. Akaike Information Criteria scores were lower for the non-zero-inflated models, and thus these were reported as planned.) A separate model was used for each research question and for each product. Analysis (1) was run on all participants. Analysis (2) was run on all participants who had used a given youth gambling product. Analysis (3) was run on all adult gamblers, and Analysis (4) was run on all adult gamblers who had also used a youth gambling product. Given these different effective sample sizes for each analysis, some inclusion criteria were preregistered. For example, if almost all participants or all adult gambler participants recollected using a given machine, then tests for hypotheses 1 and 3 would be underpowered. Instances when a model was not run for reasons of effective sample size are mentioned in the results section below, and the full inclusion criteria can be found in the preregistration.

The independent variables (frequency of engagement in each form) were coded as 0 = ‘never’, 1 = ‘seldom’, 2 = ‘occasionally’, 3 = ‘frequently’, 4 = ‘very frequently’. The response option of ‘never’ is qualitatively different to the other response options. This is why each of the two main research questions were split into independent aspects, separately looking at any level of engagement vs. no engagement (i.e., never vs. any other response; H1 and H3), and levels of engagement among those who have used a legal youth gambling product (including seldom to very frequently, but excluding never; H2 and H4).

A significance level of 0.025 was used as we introduced a Bonferroni correction to account for testing two products. A further Bonferroni correction was not introduced for splitting the overall research question into the four aspects above, as these are non-overlapping sub-components of the overall research question.

For analysis 5), we repeated all the models used for Analyses 1–4, and then added confidence scores as a main effect and as an interaction between the two- or four-value factor of rate of recollected childhood usage. Continuous independent variables were mean-centered based on the mean for the overall sample or sub-sample for each hypothesis, prior to calculating the product interaction terms. Our main outcome was to report whether any significant interactions were detected (using the same alpha threshold of 0.025). If any significant interactions were detected, then these would be interpreted to show how confidence and rates of childhood usage covary. Overall, negative interactions, whereby there are stronger associations for people who are less confident, would be the most likely to indicate that the present results are not robust to memory biases across the sample.

## Results

### Descriptives

The recollected childhood use of each machine is shown in Table 1. Most of the sample reported using both machines at least once during childhood, with recollected usage of crane grab machines (97.0%) being more common than that of coin pushers (87.2%). Participants reported high subjective confidence scores on average, of 83.3 (SD = 20.3) for coin pusher machines, and 89.2 (SD = 15.8) for crane grab machines.

**Table 1:** Recollected usage of coin pusher and crane grab machines. The first number of each cell indicated the overall average, the following numbers in parentheses represent adult non-gamblers (*n* = 987) and adult gamblers (*n* = 1,018), respectively. Total *N* = 2,005

Frequency	Coin pusher	Crane grab
Never	12.8% (16.6%, 9.0%)	3.0% (3.7%, 2.3%)
Seldom	36.3% (40.2%, 32.5%)	25.1% (30.2%, 20.1%)
Occasionally	39.0% (35.7%, 42.1%)	42.8% (43.4%, 42.2%)
Frequently	9.0% (6.1%, 11.8%)	20.3% (16.8%, 23.8%)
Very Frequently	3.0% (1.4%, 4.5%)	8.8% (5.9%, 11.6%)

### Confirmatory Analysis

The results of the models used for hypotheses 1–4 are shown in Table 2. Two of the planned analyses (H1 and H3 for crane grab machines) were not conducted as they did not meet the pre-defined inclusion criteria due to more than 95% of the relevant sub-samples recollecting using the machines in childhood, meaning that there was insufficient variance to test this outcome variable. Of the 6 associations that were tested, 5 were significant and in the hypothesized direction, as indicated by the coefficients in bold. All three of the tested associations concerning having gambled as an adult in the past 12 months were significant and in the hypothesized direction. For participants who recollected ever using coin pusher machines, the likelihood of being an adult gambler, compared to being an adult non-gambler, was 2.0 times greater (H1), and increased by 1.5 times for every one-unit increase in the usage frequency (H2). For recollected use of crane grab machines, H1 was not tested due to inclusion criteria, but the likelihood of being an adult gambler, compared to being an adult non-gambler, increased by 1.4 times per one-unit increase of childhood usage frequency (H2).

**Table 2:** Regression models predicting the gambling status (no/yes) and PGSI scores by engagement (no/yes) and frequency of engagement for coin pusher and crane grab machines. For each machine, the coefficients in the first row relate to H1 and H2 from left to right. The second row shows coefficients for H3 and H4 from left to right

Form	Variable	Statistic		Gambling status (no vs. yes)^a^		PGSI^b^
Coin pusher	Engagement (ref – no)	Coeff	**2.01**		0.91	
		95% CI	[1.53, 2.63]		[0.66, 1.25]	
		*Wald*	25.15		0.35	
		*P*	< 0.001		0.557	
		N	2,005		1,018	
	Frequency	Coeff	**1.54**		**1.41**	
		95% CI	[1.35, 1.75]		[1.26, 1.58]	
		*Wald*	43.27		36.52	
		*P*	< 0.001		< 0.001	
		N	1,749		926	
Crane Grab	Engagement (ref – no)	Coeff	Not tested due to inclusion criteria		Not tested due to inclusion criteria	
		95% CI				
		*Wald*				
		*P*				
		N				
	Frequency	Coeff	**1.44**		**1.34**	
		95% CI	[1.30, 1.60]		[1.22, 1.47]	
		*Wald*	49.59		39.13	
		*P*	< 0.001		< 0.001	
		N	1,945		995	

Note: Statistically significant coefficients are shown in bold.

^a^ Bivariate logistic regression (coefficients are odds ratios)

^b^ Negative binomial regression with log link, dispersion parameter determined by MLE (coefficients are incidence rate ratios)

For the associations with PGSI among adult gamblers, two of the three that were tested were significant and in the hypothesized direction. Recollected frequency of coin pusher machine use was significantly associated with PGSI scores amongst adult gamblers; PGSI scores increased by a factor of 1.4 per one-unit increase of recollected childhood usage (H4). The association between recollected usage of coin pusher machines, compared to not recollecting using them, and PGSI scores was non-significant (*p* = .557; H3). For crane grab machines, recollected frequency of machine use was also significantly associated with PGSI scores amongst adult gamblers; PGSI scores increased by a factor of 1.3 per one-unit increase of recollected childhood usage (H4). The association between recollected usage of crane grab machines, compared to not recollecting usage, and PGSI scores (H3) was not tested due to inclusion criteria as 97% of the sample recollected ever using them (again meaning there was insufficient variance in the dependent variable).

The results of the models used to test H5 are shown in Table 3. In these analyses, the same models were run as those for hypotheses 1–4, but with a main effect for recollection confidence and the interaction between machine engagement and recollection confidence added to the models. Most importantly for the present study, none of the interaction effects were significant.

**Table 3:** Factorial models adding main effects and interactions involving confidence to the variables shown in Table 2. For each machine, the coefficients in the first row relate to H1 and H2 from left to right. The second row shows coefficients for H3 and H4 from left to right

Effect	Statistic	DV = Adult gambler (Ref = no)		DV = PGSI score (Continuous)	
		Logistic regression		Negative binomial regression	
		Coin pusher	Crane Grab	Coin pusher	Crane grab
Main effect engagement (ref = no)	Coeff	**2.006**	Not tested due to inclusion criteria	0.910	Not tested due to inclusion criteria
	(95% CI)	[1.516, 2.656]		[0.658, 1.259]	
	*Wald*	23.68		0.33	
	p	< 0.001		0.568	
Main effect confidence	Coeff	1.000		1.001	
	(95% CI)	[0.987, 1.012]		[0.986, 1.016]	
	*Wald*	0.003		0.02	
	p	0.958		0.886	
Interaction engagement * confidence	Coeff	1.003		0.994	
	(95% CI)	[0.990, 1.016]		[0.979, 1.010]	
	*Wald*	0.19		0.53	
	p	0.667		0.466	
	N	2,005		1,018	
Main effect frequency	Coeff	**1.555**	**1.471**	**1.499**	**1.397**
	(95% CI)	[1.357, 1.784]	[1.319, 1.639]	[1.332, 1.688]	[1.270, 1.537]
	*Wald*	40.04	48.49	44.84	47.32
	p	< 0.001	< 0.001	< 0.001	< 0.001
Main effect confidence	Coeff	0.999	0.999	**0.991**	**0.990**
	(95% CI)	[0.993, 1.004]	[0.992, 1.006]	[0.986, 0.996]	[0.984, 0.996]
	*Wald*	0.24	0.06	13.46	11.03
	p	0.621	0.802	< 0.001	< 0.001
Interaction frequency * confidence	Coeff	0.999	0.993	0.999	0.996
	(95% CI)	[0.991, 1.007]	[0.985, 1.001]	[0.992, 1.005]	[0.989, 1.003]
	*Wald*	0.07	2.65	0.18	1.17
	p	0.788	0.103	0.676	0.279
	N	1,749	1,945	926	995

Note: Shaded cells indicate the output used to determine hypotheses. Bold text indicates statistically significant effects. Logistic regression output are odds ratios. Negative binomial regression with log link, dispersion parameter determined by MLE (coefficients are incidence rate ratios). Coefficients and confidence intervals are reported to 3 decimal places as some rounded values are close to the null value.

### Exploratory Analysis

Unlike the UK and Australia, in the USA some gambling opportunities are restricted to people ages 21 or above, rather than 18. For example, in most states the legal age for gambling in a casino is 21, but in some states such as Washington, Oklahoma and Idaho, the legal age is 18 for casinos (Shirley, 2022). Other forms of gambling, such as bingo, lotteries and horseracing are legally accessible to people aged 18 in most states, but in a few states, the limit is 21 years, or 16 years for bingo in Oklahoma. Thus, an exploratory analysis was conducted on a sub-sample of participants aged 21 or above (78.9% of the original sample met this threshold) to more cleanly account for adult gambler status, given that some under the age of 21 may not have had the same opportunities to gamble as others. In this sub-sample, the original findings for H1-H4 remained the same, with 5 of the 6 tested associations being significant and in the hypothesized direction (see Table 4).

For H5, this sub-sample differed slightly as a significant association was found between the interaction of confidence and coin pusher frequency and likelihood to be an adult gambler (see Table 5). The interaction term was negative, and thus the relationship between frequency of engagement with crane grab machines and adult gambler status was less strong among those who were more confident. An exploratory simple slopes analysis was run with confidence held constant at 100%. This analysis found that the relationship between frequency of crane grab use and adult gambler status was still strongly statistically significant at the highest level of confidence, coefficient = 1.53 (95% CI: 1.35; 1.73), Wald = 44.98, *p* < .001. These exploratory analyses were therefore very similar to the preregistered findings.

**Table 4:** (For participants aged 21+): Regression models predicting the gambling status (no/yes) and PGSI scores by engagement (no/yes) and frequency of engagement for coin pusher and crane grab machines. For each machine, the coefficients in the first row relate to H1 and H2 from left to right. The second row shows coefficients for H3 and H4 from left to right

Form	Variable	Statistic		Gambling status (no vs. yes)^a^		PGSI^b^
Coin pusher	Engagement (ref – no)	Coeff	**2.10**		0.95	
		95% CI	[1.55, 2.84]		[0.67, 1.35]	
		*Wald*	23.08		0.09	
		*P*	< 0.001		0.768	
		N	1,582		823	
	Frequency	Coeff	**1.57**		**1.41**	
		95% CI	[1.36, 1.81]		[1.24, 1.59]	
		*Wald*	36.88		29.60	
		*P*	< 0.001		< 0.001	
		N	1,375		748	
Crane Grab	Engagement (ref – no)	Coeff	Not tested due to inclusion criteria		Not tested due to inclusion criteria	
		95% CI				
		*Wald*				
		*P*				
		N				
	Frequency	Coeff	**1.47**		**1.35**	
		95% CI	[1.31, 1.65]		[1.22, 1.50]	
		*Wald*	42.86		31.50	
		*P*	< 0.001		< 0.001	
		N	1,536		803	

Note: Statistically significant coefficients are shown in bold.

^a^ Bivariate logistic regression (coefficients are odds ratios)

^b^ Negative binomial regression with log link (coefficients are incidence rate ratios)

**Table 5:** (For participants aged 21+): Factorial models adding main effects and interactions involving confidence to the variables shown in Table 2. For each machine, the coefficients in the first row relate to H1 and H2 from left to right. The second row shows coefficients for H3 and H4 from left to right

Effect	Statistic	DV = Adult gambler (Ref = no)		DV = PGSI score (Continuous)		
		Logistic regression		Negative binomial regression		
		Coin pusher	Crane Grab	Coin pusher	Crane grab	
Main effect engagement (ref = no)	Coeff	**2.131**	Not tested due to inclusion criteria	0.980	Not tested due to inclusion criteria	
	(95% CI)	[1.558; 2.914]		[0.677; 1.420]		
	*Wald*	22.39		0.01		
	p	< 0.001		0.980		
Main effect confidence	Coeff	1.002		1.006		
	(95% CI)	[0.988, 1.017]		[0.998, 1.023]		
	*Wald*	0.11		0.38		
	p	0.746		0.540		
Interaction engagement * confidence	Coeff	1.000		0.991		
	(95% CI)	[0.984, 1.015]		[0.973, 1.009]		
	*Wald*	0.002		0.92		
	p	0.960		0.337		
	N	1,582		823	823	
Main effect frequency	Coeff	**1.584**	**1.537**	**1.476**	**1.42**	
	(95% CI)	[1.358; 1.847]	[1.349; 1.727]	[1.296; 1.681]	[1.27, 1.58]	
	*Wald*	34.32	44.98	34.53	39.64	
	p	< 0.001	< 0.001	< 0.001	< 0.001	
Main effect confidence	Coeff	0.998	0.997	**0.992**	**0.991**	
	(95% CI)	[0.992, 1.004]	[0.990; 1.005]	[0.987; 0.998]	[0.984; 0.998]	
	*Wald*	0.31	0.50	7.50	7.19	
	p	0.579	0.478	0.006	0.007	
Interaction frequency * confidence	Coeff	1.000	**0.989**	0.999	0.992	
	(95% CI)	[0.991, 1.009]	[0.980; 0.998]	[0.991, 1.006]	[0.984; 1.000]	
	*Wald*	0.001	5.18	0.15	4.02	
	p	0.971	0.023	0.700	0.045	
	N	1,375	1,536	748	803	

Note: Shaded cells indicate the output used to determine hypotheses. Bold text indicates statistically significant effects. Logistic regression output are odds ratios. Negative binomial regression with log link, dispersion parameter determined by MLE (coefficients are incidence rate ratios). Coefficients and confidence intervals are reported to 3 decimal places as some rounded values are close to the null value.

## Discussion

This study aimed to investigate the association between childhood use of coin pusher and crane grab machines, and adult gambling participation and problems in a young adult USA population. Two novel findings were that most of the USA-based participants recollected using these machines at least once in their childhood (87.2% for coin pushers and 97.0% for crane grab machines), and that participants had high subjective confidence ratings of these memories (83.3 and 89.2 respectively). Overall, 5 of the 6 tested associations to be statistically significant and all in their hypothesized direction. The only non-significant association found in our study concerned H3, as PGSI scores were not found to be significantly associated with having ever engaged with a coin pusher machine during childhood. However, higher frequencies of recollected coin pusher machine usage were significantly associated with higher PGSI scores. These results are mostly similar with those of the previous study (Parrado-González & Newall, 2022), which reported 5 out of 7 of their tested hypotheses to be statistically significant and in their hypothesized direction. However, the association found to be non-significant in our study cannot be directly compared with this prior research, as that was the one hypothesis failing the inclusion criteria in that study.

Tests of H5 suggested that these findings were largely unaffected by interactions with subjective confidence. If associations between legal youth gambling and adult gambling status or problems were only found amongst those with low levels of subjective confidence, but not those with high confidence, this may have raised concerns about the findings. However, this was not the case, and interaction effects were largely non-significant. The single significant interaction found that while the relationship between frequency of use of crane grab machines and adult gambler status was stronger amongst those who were less confident, it was still statistically significant among those who indicated confidence of 100%. Thus, it appears that the relationship was still observed at different levels of confidence, reducing concerns around memory effects.

The exploratory analyses on the subsample aged 21 and over were run as, in the USA, gambling age restrictions vary by state and by form of gambling. For example, in most states, access to casinos is restricted to people aged 21 years or above, however, in some states such as Washington, Oklahoma or Idaho, these are legally accessible by those aged 18 years or over. However, other forms of gambling, such as lotteries or horseracing are legally accessible by those aged 18 or above in most states, so it is plausible most USA residents ages 18 or above would have access to some form of legal gambling. While most of our sample was aged 21 or over (78.9% of the sample), we ran an exploratory analysis only on participants aged 21. This involved repeating the analyses for H1-H5 with a sub-sample of only the participants who were aged 21 years or older. Results from this exploratory analysis were mostly the same as the confirmatory analysis, as five out of the six tested hypotheses remained significant and in their hypothesized direction. This suggests that the risk of participants not being able to legally gamble at the time of participation was likely minimal and that these associations are still valid as they remain significant in a sample which met age requirements to legally gamble, regardless of state. The one significant interaction here, between frequency and confidence for the likelihood of being an adult gambler when recollecting childhood crane grab use may reflect a difference between the machines, as the same interaction was not found to be significant for recollected childhood coin pusher use. This may reflect physical differences between the childhood gambling machines, such as appearance characteristics that made recollections more salient, or physical availability which could have influenced use frequency. However, the coefficients and confidence intervals were very close between the two machines, so it is possible that any differences here are marginal, but further research is required to confirm this difference between them.

The findings of this research have implications for the consideration of policy concerning legal childhood gambling machines. As these associations have been repeatedly demonstrated, across countries and replications (Newall et al., 2021; Parrado-González & Newall, 2022), it suggests that the childhood use of legal gambling machines may be a risk factor for later gambling disorders as an adult. More jurisdictions should consider adding questions about these machines to their youth gambling surveys, as the UK’s Gambling Commission (2022) recently did. High engagement rates with these products, compared to much lower rates of engagement with age-restricted gambling products, should make further research on coin pusher and crane grab machines of relevance to a public health approach to reducing youth gambling harms.

These findings are subject to various limitations. Although the sample was young, and therefore there was expected to be fewer memory issues about their adolescence, recollection biases could still have influenced these results. Longitudinal research tracking participants as they move from childhood to young adulthood are therefore recommended. The results are also correlational and not causal. Observed associations may be driven by unobserved third factors, such as high trait impulsivity, which affects both childhood gambling behaviours and later adult gambling (Chambers & Potenza, 2003). The sample was recruited from a crowdsourcing platform, and so was not representative of the general population (Mishra & Carleton, 2017). Further research should therefore be conducted in for example representative gambling prevalence surveys.

In conclusion, this study extended prior literature on coin pusher and crane grab machines to the USA, by finding that most young adults recollected using them during their childhood, that this behaviour was correlated with their adult gambling, and that these findings were largely robust to potential concerns around subjective confidence. Future research should build upon this research to investigate if these associations are present in more countries and explore the extent to which usage of these machines has any causal effect on gambling harm across the lifespan.
